# High patient satisfaction with a simplified *BRCA1*/*2* testing procedure: long-term results of a prospective study

**DOI:** 10.1007/s10549-018-5000-y

**Published:** 2018-10-11

**Authors:** Martin P. Nilsson, Erik D. Nilsson, Åke Borg, Yvonne Brandberg, Barbro Silfverberg, Niklas Loman

**Affiliations:** 1 Division of Oncology and Pathology, Department of Clinical Sciences, Lund University, Lund, Sweden; 2 Department of Hematology, Oncology and Radiation Physics, Skåne University Hospital, Lund, Sweden; 3 Clinical Memory Research Unit, Department of Clinical Sciences Malmö, Lund University, Lund, Sweden; 4 Department of Oncology-Pathology, Karolinska Institutet, Stockholm, Sweden; 5 Department of Clinical Genetics, Laboratory Medicine Region Skåne, Lund, Sweden

**Keywords:** BRCA1, BRCA2, Breast cancer, Simplified, Genetic counseling, Satisfaction

## Abstract

**Purpose:**

In the BRCAsearch study, unselected breast cancer patients were prospectively offered germline *BRCA1*/*2* mutation testing through a simplified testing procedure. The purpose of the present study was to evaluate satisfaction with the BRCAsearch testing procedure and, furthermore, to report on uptake rates of prophylactic surgeries among mutation carriers.

**Methods:**

Pre-test information was provided by a standardized invitation letter instead of in-person genetic counseling. The patients were offered contact with a genetic counselor for telephone genetic counseling if they felt a need for that. Mutation carriers were telephoned and given a time for a face-to-face post-test genetic counseling appointment. Non-carriers were informed about the test result through a letter. One year after the test results were delivered, a study-specific questionnaire was mailed to the study participants who had consented to testing. The response rate was 83.1% (448 of 539).

**Results:**

A great majority (96.0%) of the responders were content with the method used for providing information within the study, and 98.7% were content with having pursued genetic testing. 11.1% answered that they would have liked to receive more oral information. In an adjusted logistic regression model, patients with somatic comorbidity (OR 2.56; *P* = 0.02) and patients born outside of Sweden (OR 3.54; *P* = 0.01) were more likely, and patients with occupations requiring at least 3 years of university or college education (OR 0.37; *P* = 0.06) were less likely to wanting to receive more oral information. All 11 mutation carriers attended post-test genetic counseling. At a median follow-up of 2 years, the uptake of prophylactic salpingo-oophorectomy was 100%, and the uptake of prophylactic mastectomy was 55%.

**Conclusions:**

Satisfaction with a simplified *BRCA1*/*2* testing procedure was very high. Written pre-test information has now replaced in-person pre-test counseling for breast cancer patients in our health care region.

**Electronic supplementary material:**

The online version of this article (10.1007/s10549-018-5000-y) contains supplementary material, which is available to authorized users.

## Introduction

Simplified methods, such as telephone counseling or written information, are increasingly used instead of standard face-to-face pre-test genetic counseling [1–5]. We have recently reported the results of the prospective BRCAsearch study, where unselected breast cancer patients were offered germline *BRCA1* and *BRCA2* mutation testing [6]. Instead of pre-test genetic counseling, the patients received a standardized information letter. The study procedure offers an example of how genetic testing could be undertaken on a large scale, enabling BRCA testing to be expanded to a much larger number of patients than what has previously been possible.

Studies on patient satisfaction with genetic counseling have reported high levels of satisfaction, regardless if the counseling was conducted in-person, by telephone, or if the information was primarily provided through written material [7–11]. To the best of our knowledge, no previous study has reported on patient satisfaction with written pre-test written information offered to unselected breast cancer patients. In this paper, we present the results of the 1-year follow-up patient-reported questionnaire from the BRCAsearch study. The questionnaire included questions regarding satisfaction with the study procedure and genetic testing.

The mode of delivery of genetic counseling and testing services could be associated with uptake rates of cancer risk management strategies among mutation carriers [12]. Importantly, prophylactic surgeries in *BRCA1*/*2* mutation carriers lead to a significant survival benefit [13]. It is therefore crucial to rule out any negative impact on the uptake rates of prophylactic surgeries before new testing procedures are implemented in daily practices. In this paper, we also present the uptake rates of prophylactic surgeries among the mutation carriers identified within the BRCAsearch study.

## Materials and methods

### The *BRCAsearch* study

The study population of BRCAsearch has been described in detail elsewhere [6, 14]. Briefly, patients newly diagnosed with breast cancer were prospectively offered germline *BRCA1* and *BRCA2* mutation testing, unselected for age at diagnosis or family history of cancer (BRCAsearch, ClinicalTrials.gov Identifier: NCT02557776). Pre-test information was provided by a standardized invitation letter instead of in-person genetic counseling. The patients were offered contact with a genetic counselor for telephone genetic counseling if they felt a need for that. As previously reported, the invitation letter was given to 818 patients during February 2015–August 2016. One patient only had lobular carcinoma in situ, and she was therefore excluded from further analyses. Twelve patients did not consent to further follow-up, and were also excluded. Among the remaining 805 patients, 539 (67.0%) consented to germline testing. Only 11 out of 539 tested patients contacted us for questions related to genetic counseling [6]. Mutation carriers were telephoned and given a time for a face-to-face post-test genetic counseling appointment within 1 week. Non-carriers were informed about the test result through a letter.

### Present study population and study procedure

One year after delivery of the test results, a study-specific questionnaire was mailed to participants who had consented to testing. The questionnaire included 6 questions related to satisfaction with the study procedure and genetic testing (Table 1). The response rate was 83.1% (448 of 539). Responders were older than non-responders (mean age: 62.5 vs. 59.1 years; *P* = 0.01), and less likely to be born outside of Sweden (6.9 vs. 15.4%; *P* = 0.008) (Supplementary table S1). Responders (*n* = 448) constituted the study population for the present paper. Patients who did not elect to participate in the BRCAsearch study, and as a consequence were not tested, were not part of this follow-up.

**Table 1:** Questions and answers of the 1-year follow-up questionnaire

Question	Answer		
	“To a high extent” or “To a very high extent” *n* (%)	“Not at all” or “To a low extent” *n* (%)	Missing answer
Q1: Did you understand the information that was provided within the study?	434 (97.1)	13 (2.9)	1
Q2: Would you have liked to receive more oral information?	49 (11.1)	394 (88.9)	5
Q3: Are you content with having pursued genetic testing?	441 (98.7)	6 (1.3)	1
Q4: Are you content with the method used for providing information within the study (written information with the possibility of further contact)?	428 (96.0)	18 (4.0)	2
Q5: Would you recommend a female friend with breast cancer to pursue genetic testing in the way that you have done?	437 (97.8)	10 (2.2)	1
Q6: Have you shared the information that you have received with your relatives?	321 (71.7)	127 (28.3)	0

### Data collection

As detailed in a previous paper [14], the clinical data were abstracted from the medical records. Different occupations were categorized into three groups based on the minimum level of education required. Group 1 consisted of occupations requiring little formal education other than compulsory school. Occupations requiring some, but less than 3 years, of vocational school or college education were allocated to Group 2. Group 3 consisted of occupations requiring at least 3 years of college or university education, equivalent to a bachelor’s degree or a master’s degree. Information about somatic comorbidity was coded according to the Charlson comorbidity index (CCI) [15]. A recent diagnosis of breast cancer rendered 2 points, and accordingly, no patient had a CCI of less than 2. Age categories are sometimes included in the CCI, but since we aimed to include age as a separate variable in the logistic regression model, CCI in this paper refers to CCI excluding age.

### Statistical analyses

Differences in patient characteristics between patients who returned the questionnaire and patients who did not return the questionnaire were assessed using Pearson Chi-square test (*χ*^2^) and independent samples *t* test. Regarding predictors of wanting to receive more oral information (Table 2), unadjusted associations were evaluated using logistic regression. In the multivariable logistic regression model, variables with a significance of *P* ≤ 0.25 in unadjusted analysis were included. All analyses were conducted using SPSS version 22.0 (SPSS Inc., Chicago, Illinois, USA).

**Table 2:** Logistic regression models assessing predictors of wanting to receive more oral information

Variable	Crude OR	95% CI	*P* value	Adjusted OR^a^	95% CI	*P* value
Age, categories						
< 50 years	ref			ref		
50–59 years	1.19	0.58–2.44	0.63	1.78	0.52–6.07	0.36
60–69 years	1.53	0.84–2.79	0.17	2.28	0.72–7.25	0.16
70–79 years	0.69	0.32–1.47	0.34	0.99	0.22–4.39	0.99
≥ 80 years	1.91	0.53–6.96	0.33	3.12	0.23–41.91	0.39
Occupation, categories						
1	ref			ref		
2	1.46	0.73–2.91	0.28	1.09	0.51–2.32	0.83
3	0.28	0.11–0.73	0.01	0.37	0.13–1.03	0.06
Psychiatric disease						
Yes versus No	0.42	0.10–1.81	0.25	0.58	0.13–2.61	0.47
CCI						
≥ 3 versus 2	2.00	1.00–4.00	0.05	2.56	1.15–5.72	0.02
Country of birth						
Outside of Sweden versus Sweden	3.81	1.64–8.82	0.002	3.54	1.34–9.37	0.01
Children						
Yes versus No	0.82	0.35–1.94	0.65			
Previous BC						
Yes versus No	0.64	0.22–1.86	0.41			
BC or OC in FDR or SDR						
Yes versus No	0.70	0.34–1.43	0.33			

*OR* odds ratio, *CI* confidence interval, *ref* reference, *BC* breast cancer, *OC* ovarian cancer, *FDR* first degree relative, *SDR* second degree relative, *CCI* Charlson comorbidity index (excluding age)

^a^Multivariable logistic regression including age, occupation, psychiatric disease, CCI, and country of birth

## Results

### Satisfaction with testing and with the study procedure

Overall, the study participants were satisfied with the study procedure (Table 1). 97.1% expressed that they had understood the information provided within the study, and 96.0% were content with the method used for providing information within the study. 97.8% would recommend a female friend with breast cancer to pursue genetic testing in the way that they had done, and 98.7% were content with having pursued genetic testing. On the question “Would you have liked to receive more oral information,” a majority (88.9%) answered “Not at all” or “To a low extent,” but 11.1% (*n* = 49) responded “To a high extent” or “To a very high extent.” Still, out of 49 patients who answered that they wanted to receive more oral information, only 5 had contacted us for questions, and 45 out of 49 reported that they were content with the method used for providing information within the study.

Exploratory analyses were conducted to investigate if any patient characteristics were associated with a wish to receive more oral information (Table 2). In the adjusted logistic regression model, patients with somatic comorbidity (OR 2.56; *P* = 0.02) and patients born outside of Sweden (OR 3.54; *P* = 0.01) were more likely, and patients with occupations requiring at least 3 years of university of college education (OR 0.37; *P* = 0.06) were less likely to wanting to receive more oral information.

Within the present study population, 29 patients had contacted us for questions, and consequently, they had received pre-test telephone genetic counseling in addition to the written information provided in the invitation letter. Only 3 out of 29 answered “Not at all” or “To a low extent” on the question “Are you content with the method used for providing information within the study (written information with the possibility of further contact).” The remaining 26 were content with the method.

### Mutation carriers

Eleven germline *BRCA1*/*2* mutation carriers were identified within the BRCAsearch study (*BRCA1, n* = 2; *BRCA2, n* = 9) [6]. All of them attended in-person post-test genetic counseling. At a median follow-up of 2 years, one mutation carrier had been diagnosed with a local recurrence following breast-conserving surgery; all other mutation carriers were free of breast cancer recurrence. Eleven (100%) had undergone prophylactic salpingo-oophorectomy, and six (55%) had undergone prophylactic mastectomy.

All mutation carriers were content with having pursued genetic testing, and 10 out of 11 were content with the method used for providing information within the study. One of them would have liked to receive more oral information.

## Discussion

We offered a simplified and streamlined *BRCA1*/*2* testing protocol to unselected breast cancer patients. Among the patients who opted for testing (67.0%), satisfaction with the testing procedure was very high. Also within the small group of patients who turned out to be mutation carriers (2.0%), there was a high degree of satisfaction with the testing procedure.

To the best of our knowledge, our study is the first to assess satisfaction in unselected breast cancer patients who have undergone germline *BRCA1*/*2* testing without prior face-to-face genetic counseling. Similar to the results of our study, previous studies in cohorts enriched for mutation carriers and cohorts from clinical genetics departments have reported high levels of satisfaction with the standard procedure of in-person counseling, with telephone counseling, and with simplified methods based on written material [8, 9, 11]. Metcalfe et al. found a high rate of satisfaction (92.8%) with the testing procedure among unselected Jewish women who underwent *BRCA1*/*2* testing without pre-test genetic counseling [7]. One could conclude that a great majority of all patients seem to be satisfied with any type of delivery of *pre-test* information. Given that

- proper *post-test* genetic counseling carried out by a genetics professional is important for the uptake rates of prophylactic surgeries [12],
- mutation carriers who are aware of their mutation carrier status—in contrast to mutation carriers who are not aware of their mutation carrier status—can opt for prophylactic surgeries or surveillance programs, thereby decreasing their risk of cancer-related death [16],
- currently used testing procedures based on selection criteria to merit testing fail to detect up to half of the mutation carriers [17–20],

it seems prudent to recommend simplified methods for pre-test information and, instead, let the scarce resource of genetics professionals focus on the post-test genetic counseling for the small minority of cancer patients who will turn out to be mutation carriers. In our opinion, the results of our present study lend support to such an approach. Importantly, all *BRCA1*/*2* mutation carriers identified within the study accepted the offer of in-person post-test genetic counseling, and with only 2 years of median follow-up, all of them had opted for prophylactic salpingo-oophorectomy.

A minority of breast cancer patients offered germline *BRCA1*/*2* testing would likely benefit from pre-test counseling—telephone or in-person—as a complement to written information. In our study, 11.1% reported that they would have liked to receive more oral information. Using data extracted from the medical records, we were able to identify some characteristics associated with a higher likelihood of wanting more oral information: patients with somatic comorbidity and patients born outside of Sweden were more likely, and patients with a high level of education were less likely to wanting to receive more oral information. However, we do not believe that the results of these exploratory analyses should have any clinical impact. When a standardized written pre-test information is used, we consider it very important to offer all patients the possibility of complementary telephone or in-person counseling, and consider it mandatory to offer all mutation carriers post-test in-person genetic counseling.

For many breast cancer patients, simplified methods might even be preferable over standard pre-test counseling. In the “DNA-direct” study from the Netherlands, Sie et al. found that some patients, who were still undergoing breast cancer treatment, opted for simplified pre-test information because an extra hospital visit in a time period of chemotherapy or radiotherapy treatment was considered an extra burden, while reading information at home made genetic testing accessible [5].

There are limitations to our study. First, we used a questionnaire that was developed for the study. The reason for this was that we did not find any validated questionnaire fulfilling our requirements for evaluation of the quite specific study procedure. The questions and answers (translated from Swedish to English) of our questionnaire are presented in Table 1, making it possible for the reader to assess the utility of the questionnaire. Second, there is the issue of representativity. A response rate of 83.1% is good for a mailed 1-year follow-up questionnaire. Still, one important aspect to consider is that all the patients who were mailed the questionnaire had opted for genetic testing. Such patients are obviously biased towards a positive attitude to genetic testing in general. It is not surprising that they report a high level of satisfaction. Due to ethical regulations, we were not able to survey patients who were offered, but did not pursue, genetic testing (266 of 805; 33%), and consequently, we cannot assess whether those patients were satisfied with—or even read—the written information that was provided.

In summary, satisfaction with a simplified testing procedure was very high among unselected breast cancer patients undergoing germline *BRCA1*/*2* testing. Based on the results of the BRCAsearch study, in conjunction with the similar results of other studies, written pre-test information has replaced in-person pre-test counseling for recently diagnosed breast cancer patients in our health care region. As a consequence, germline *BRCA1*/*2* testing can now be offered to a much larger number of breast cancer patients than what previously was possible.

## Electronic supplementary material

Below is the link to the electronic supplementary material.

Supplementary material 1 (DOCX 22 KB)
