# Supplementary material for: High patient satisfaction with a simplified BRCA1/2 testing procedure: long-term results of a prospective study
Source: Breast Cancer Res Treat. 2018 Oct 11;173(2):313–8. doi: 10.1007/s10549-018-5000-y (PMC6394590; doi:10.1007/s10549-018-5000-y)
Supplement: Supplementary file 1 — Supplementary material 1 (DOCX 22 KB) [file 10549_2018_5000_MOESM1_ESM.docx]

| **Table S1**. Comparison between patients who returned the questionnaire and patients who did not return the questionnaire | | | |  |
| --- | --- | --- | --- | --- |
|  | **Returned the questionnaire (*n* = 448)** | **Did not return the questionnaire (*n* = 91)** |  |  |
| **Variable** | ***n* (%)** | ***n* (%)** | ***P*-value**^a^ |  |
| Age at diagnosis (years) |  |  |  |  |
| Mean; SD | 62.5; 11.4 | 59.1; 13.1 | 0.01^b^ |  |
| Age at diagnosis, categories |  |  | 0.005 |  |
| <50 years | 74 (16.5) | 29 (31.9) |  |  |
| 50-59 years | 90 (20.1) | 19 (20.9) |  |  |
| 60-69 years | 159 (35.5) | 26 (28.6) |  |  |
| 70-79 years | 108 (24.1) | 12 (13.2) |  |  |
| ≥80 years | 17 (3.8) | 5 (5.5) |  |  |
| Occupation, categories^c^ |  |  | 0.32 |  |
| 1 | 160 (41.8) | 26 (33.3) |  |  |
| 2 | 104 (27.2) | 22 (28.2) |  |  |
| 3 | 119 (31.1) | 30 (38.5) |  |  |
| Missing | 65 | 13 |  |  |
| Children |  |  | 0.47 |  |
| No | 56 (12.6) | 9 (9.9) |  |  |
| Yes | 388 (87.4) | 82 (90.1) |  |  |
| Missing | 4 |  |  |  |
| Previous BC^d^ |  |  | 0.32 |  |
| No | 395 (88.4) | 77 (84.6) |  |  |
| Yes | 52 (11.6) | 14 (15.4) |  |  |
| Missing | 1 |  |  |  |
| BC *or* OC in FDR *or* SDR |  |  | 0.26 |  |
| No | 286 (67.9) | 55 (61.8) |  |  |
| Yes | 135 (32.1) | 34 (38.2) |  |  |
| Missing | 27 | 2 |  |  |
| Psychiatric disorder |  |  | 0.16 |  |
| No | 409 (91.5) | 79 (86.8) |  |  |
| Yes | 38 (8.5) | 12 (13.2) |  |  |
| Missing | 1 |  |  |  |
| Charlson comorbidity index (CCI) |  |  | 0.81 |  |
| CCI = 2 | 373 (83.4) | 75 (82.4) |  |  |
| CCI ≥ 3 | 74 (16.6) | 16 (17.6) |  |  |
| Missing | 1 |  |  |  |
| County of birth |  |  | 0.008 |  |
| Outside of Sweden | 31 (6.9) | 14 (15.4) |  |  |
| Sweden | 417 (93.1) | 77 (84.6) |  |  |
| Abbreviations: SD, standard deviation; BC, breast cancer; OC, ovarian cancer; FDR, first degree relative; SDR, second degree relative | | | | |
| ^a^ Pearson Chi-square for all if otherwise not noted | |  |  |  |
| ^b^ Independent samples *t*-test |  |  |  |  |
| ^c^ See Material and Methods section for definitions of categories | | |  |  |
| ^d^ Previous breast cancer (invasive or ductal carcinoma in situ) in the ipsilateral or in the contralateral breast | | | | |
